# Novel Cellulose Nanocrystals-Based Polyurethane: Synthesis, Characterization and Antibacterial Activity

**DOI:** 10.3390/polym14112197

**Published:** 2022-05-28

**Authors:** Maolan Zhang, Xiujuan Lu, Guiping Zhang, Xiaoling Liao, Jiale Wang, Na Zhang, Chunyi Yu, Guoming Zeng

**Affiliations:** 1 Chongqing Engineering Laboratory of Nano/Micro Biological Medicine Detection Technology, Chongqing University of Science and Technology, Chongqing 401331, China; zml@cqu.edu.cn (M.Z.); 2020202038@cqust.edu.cn (X.L.); 2018441607@cqust.edu.cn (G.Z.); zxc_228@163.com (X.L.); 2019440721@cqust.edu.cn (J.W.); 2019440880@cqust.edu.cn (N.Z.); 2 Department of Construction Management and Real Estate, Chongqing Jianzhu College, Chongqing 400072, China; 3 School of Civil Engineering and Architecture, Chongqing University of Science and Technology, Chongqing 401331, China

**Keywords:** cellulose nanocrystals, polyurethane, tannic acid, Ag NPs, antibacterial activity

## Abstract

As a new type of polymer, water-driven polyurethane (PU) has attracted increasing attention of researchers; however, with the popularization of its application, the following infection problems limit their applications, especially in the biomedical field. Herein, a series of novel cellulose nanocrystals (CNCs)-based PUs were first synthesized by chemical cross-linking CNCs with triblock copolymer polylactide–poly (ethylene glycol)–polylactide (CNC-PU). After covalent binding with tannic acid (TA-CNC-PU), the silver nanoparticles (Ag NPs) were further introduced into the material by a reduction reaction (Ag/TA-CNC-PU). Finally, the prepared serial CNCs-based PU nanocomposites were fully characterized, including the microstructure, water contact angle, water uptake, thermal properties as well as antibacterial activity. Compared with CNC-PU, the obtained TA-CNC-PU and Ag/TA-CNC-PU were capable of lower glass transition temperatures and improved thermal stability. In addition, we found that the introduction of tannic acid and Ag NPs clearly increased the material hydrophobicity and antibacterial activity. In particular, the Ag/TA-CNC-PU had a better antibacterial effect on *E. coli*, while TA-CNC-PU had better inhibitory effect on *S. aureus* over a 24 h time period. Therefore, these novel CNCs-based PUs may be more beneficial for thermal processing and could potentially be developed into a new class of smart biomaterial material with good antibacterial properties by adjusting the ratio of TA or Ag NPs in their structures.

## 1. Introduction

As the population ages, the number of patients with difficult diseases increases and the development of high-tech, biomedical materials progresses fast. Among these developments, biomedical polyurethane (PU) is a kind of biodegradable polymer, which has been widely used in the biomedical fields in various capacities, such as dressings, sutures and catheters, due to its good biocompatibility, non-toxicity and designable capability of molecules [1,2,3]. For example, the material properties of PU depend on the type of chain extender and polyol and the extent of cross-linking as well as the chain length of the polyol, where a rigid structure or high degree of cross-linking yields a rigid polyurethane, and long chain polyols yield flexible PU. In recent years, with the proposal of “intelligent” materials, shape memory polyurethane has gradually emerged [4,5,6,7]. By designing the type and content of a hard segment and soft segment, a cross-linking agent and a filler doping method, the obtained PU can respond to environmental changes, i.e., temperature, light, electromagnetic, magnetism and solvent [8,9]. Water-driven PUs, especially, have received increasing amounts of attention in the biomedical field because this stimulus is mild and friendly [10,11]. For example, Jafari et al. introduced cellulose nanocrystals (CNCs) into linear PUs, which, based on poly (ethylene glycol)-poly (ε-caprolactone), blends to prepare hydrogel PU, and the obtained compound material could well respond to the tissue fluid [12]. In addition, the combination of CNCs and PU can aid in the development of bionanocomposites containing a significant amount of renewable carbon content [13]. Current reports on CNCs in PU film mainly focus on improving the interaction between the nanofillers and the polymer molecular chains so as to enhance the thermal properties, material wettability and mechanical properties of the polymer [14,15,16,17]. However, medical materials are foreign substances in the human body and, with their widespread applications in clinic practice, related infections have become one of the more common complications [18,19]. In order to effectively solve this problem, the current study mainly focused on developing antibacterial adhesion or bactericidal materials [20,21,22]. For instance, silver nanoparticles (Ag NPs) are considered as a broad-spectrum bactericide exhibiting bacteriostatic activity and other functions, such as anti-inflammatory activities at low concentrations [23,24]. The incorporation of Ag NPs in various materials can be appropriately engineered to suit different medical application scenarios, such as wound healing [25], tissue engineering [26] and prevent bacterial adhesion on medical devices [27]. For example, Shi et al. prepared antibacterial polyurethane-g-polyethylene glycol (TPU-g-PEG)/Ag NPs nanofiber composites, and the obtained materials exhibited excellent hemocompatibility, in particular, better antibacterial properties [28]. Therefore, the water-induced shape memory composites with antibacterial properties in this study are especially attractive for potential biomedical applications, such as self-tightening sutures and adaptive smart dressings.

Herein, we first adopted the strategy of chemical cross-linking to introduce CNCs into the molecular structure of triblock copolymer polylactide–poly (ethylene glycol)–polylactide (PLLA-PEG-PLLA) to prepare water-driven PU. Both PLLA and PEG have been widely applied as biomaterials in medicine because of their good biocompatibility. The end hydroxyl groups of PLLA-PEG-PLLA were first functionalized with 1,6-hexamethylene diisocyanate (HDI), and then followed by chain extension with the hydroxyl groups of CNCs. Then, tannic acid (TA) was grafted onto the above CNCs-based PU to prepare a novel carrier (TA-CNC-PU). TA is a naturally occurring polyphenol with a high density of pyrogallic acid or catechol groups on its structure [29]. The ortho phenolic hydroxyl groups in TA can form stable five-membered chelating rings with metal ions and also have strong reduction ability for Ag^+^, etc. [30]. In view of this, we further introduced Ag NPs into CNCs-based PU materials in this way. This strategy could provide a potential platform for enhancing the thermal properties and material wettability of PUs. Furthermore, the nanocomposite network exhibited antibacterial functions. The advantages of these CNCs-based PUs make them an attractive candidate for potential applications as biomedical materials and environmentally friendly materials.

## 2. Materials and Methods

### 2.1. Materials

Cellulose micro-crystalline (MCC, Mn = 162.06), TA (ACS reagent), PEG (average Mn 200), glutaraldehyde and silver nitrate (AgNO_3_) were purchased from Aladdin Biochemical Technology Co., Ltd. (Shanghai, China). CNCs were prepared from MCC by hydrolysis treatment with few modifications [31]. HDI (99% purity) and stannous octoate (Sn(Oct)_2_, 95% purity) were purchased from Sigma-Aldrich Chemical Co. (Shanghai, China). L-lactide (L-LA, 99.5% purity) was purchased from Daigang Biomaterial Co. (Jinan, China). Sulfuric acid (H_2_SO_4_), anhydrous ethanol, chloroform, N,N-Dimethylformamide (DMF) and anhydrous toluene were purchased from Chongqing Drug Stock Limited Co. (Chongqing, China). Escherichia coli (*E. coli*, ATCC 25922) and Staphylococcus aureus *(S. aureus*, ATCC 6538), obtained from the Beijing Centers for Disease Prevention (Beijing, China), were used as the model prokaryotic organisms in this research. They were first cultured in Luria–Bertani (LB) agar and grown aerobically at 37 °C until the mid-logarithmic phase of growth. For the following antibacterial activity tests, a starting concentration of ~10^6^ CFU/mL was needed. Deionized water was made in the laboratory.

### 2.2. Synthesis of CNC-PU

PU nanocomposites composed of HDI, PLLA-PEG-PLLA diols and CNCs as chain extenders were synthesized by a two-step process. Firstly, the PLLA-PEG-PLLA diols were synthesized by a ring-opening polymerization of L-lactide in the presence of Sn(Oct)_2_ as an initiator and PEG 200 as a co-initiator. Subsequently, a set proportion of PLLA-PEG-PLLA diols, HDI, Sn(Oct)_2_ and anhydrous toluene were added into a 100 mL three-necked round flask equipped with a magnetic stirrer and the reaction was continued at 75 °C for 3 h under a flowing nitrogen atmosphere. Finally, the stable CNCs solution in DMF (pre-ultrasonic dispersion) was added dropwise into the above reaction system for chain extension at 80 °C for another 4 h. The products were purified by ethanol aqueous solution and then dried under vacuum at room temperature for at least 48 h.

### 2.3. Preparation of TA-CNC-PU

A specific proportion of CNC-PU and TA was first added to anhydrous DMF solution for a stirring reaction at 25 °C for 6 h. Then, a pre-set ratio of glutaraldehyde solution (volume fraction of 2 %, pH = 6.0 to 6.5) was added dropwise with continuous stirring to react for another 6 h at 35 °C. The TA-CNC-PU materials were finally obtained after being dialyzed (molecular weight cutoff = 3500 Da) with DI water for 3 days and lyophilized.

### 2.4. Synthesis of Ag/TA-CNC-PU

**Figure 1:**
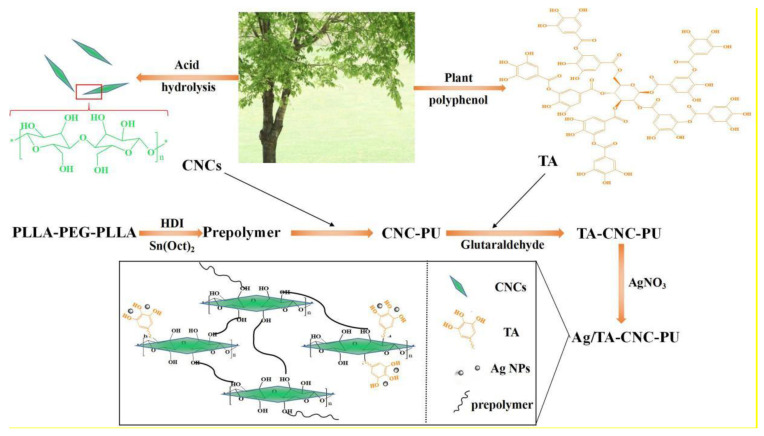
The preparation process of Ag/TA-CNC-PU.

The Ag/TA-CNC-PU nanocomposites were prepared by mixing TA-CNC-PU and AgNO_3_ in appropriate stoichiometric concentrations under room temperature for 6 h. The obtained suspension was then dialyzed (molecular weight cutoff = 3500 Da) with DI water for 3 days and lyophilized for the subsequent characterization. The specific synthesis path is presented in Figure 1.

### 2.5. Structural Characterization

The Fourier transform infrared (FT-IR) spectroscopy analysis of CNC-PU, TA-CNC-PU and Ag/TA-CNC-PU was carried out on a Perkin Elmmer Spectrum GX model (Perkin Elmer Inc., Waltham, MA, USA), and the detection range was from 400 to 4000 cm^−1^. X-ray photoelectron spectroscopy (XPS) measurements were carried out on a Thermo ESCALAB 250Xi electron spectrometer (Thermo Fisher Scientific, Waltham, MA, USA)to analysis the binding energy spectrum of C1s, O1s, N1s and Ag3d on the surface of CNCs-based PUs films, and the detection range was from 0~1200 eV. Moreover, the auger spectrum was also chosen to determine the valence state of Ag in Ag/TA-CNC-PU. The structure of CNCs was investigated using a JEM 1200EX transmission electron microscopy (JEOL Ltd., Japan). The CNCs suspension was dispersed evenly with an ultrasonic cell grinder and then observed under the TEM after negative staining with phosphotungstic acid. Ag/TA-CNC-PU was first dissolved thoroughly in the mixture of chloroform and DMF (V_CHCl3_:V_DMF_ = 9:1) and then cast as films. The surfaces of the films were investigated using an SU8020 scanning electron microscopy (HITACHI Ltd., Tokyo, Japan) after drying and gold spraying.

### 2.6. Thermal Analysis

The thermal stability of CNCs-based PUs was characterized using a STA449C thermal analysis system (NETZSCH Co., Bavaria, Germany). About 10 ± 0.1 mg of materials were prepared, and the test were performed under an Ar atmosphere with a gas flow and was heated up from ambient temperature to 500 °C. The heating rate of 10 °C/min was chosen, and the changes of the CNCs-based PUs’ weight with temperature were recorded continuously.

The series CNCs-based PUs were measured from two cyclic heating and cooling scans (−20 °C to 120 °C) by a differential scanning calorimeter (Perkin Elmer Inc., Waltham, MA, USA) under a dry nitrogen gas flow, using 5–6 mg samples. The DSC measurement was performed at a heating and cooling rate of 10 °C min^−1^.

### 2.7. Characterization of Hydrophilic and Hydrophobic Properties

Contact angle equipment (DSA 100, KRUSS Co., Hamburg, Germany) was used to measure the hydrophilic properties of CNCs-based PU films at room temperature. A drop of deionized water (5 μL) was applied manually to the coating surface, and the resulting contact angle was measured (all contact angle data were the averages of five measurements obtained at different locations on the sample surfaces).

The water uptake of CNC-PU, TA-CNC-PU and Ag/TA-CNC-PU membrane materials were weighed before and after immersion in deionized water at 37 °C for different time durations, and the weight of the sample before immersion in water was recorded as *W*_0_; *W*_t_ refers to the weight of the sample after immersion in water at 37 °C for different time durations. The water absorption ratio of CNCs-based PU films was calculated according to the following formula.

(1) $$\mathrm{Water}\;\mathrm{absorption}\;\mathrm{ratio}=\frac{W_{t}-W_{{}_{0}}}{W_{{}_{0}}}\;\times \;100\%$$

### 2.8. Antibacterial Activity Tests

The in vitro antibacterial activity of CNCs-based PUs against *E.coli* and *S.aureus* was investigated by the disc diffusion method. Firstly, *bacterial* suspension was evenly spread on the surface of LB solid medium using sterile cotton swabs. Drug sensitive papers containing different materials were placed onto the inoculated LB solid medium surface and incubated at 37 °C for 18 h. After incubation, the inhibition zones around the drug sensitive papers were imaged using a digital camera. According to the literature, log reduction in CFU counts represents an antimicrobial action [32,33], so the bacteriostatic rate test was also employed to further evaluate the antibacterial activity of CNCs-based PUs. To be specific, a series of CNCs-based PU films were first cut into a 4 mm × 4 mm standard size and then sterilized by UV. After that, these samples with a specific weight were added to the shake tubes and then 10 mL of *bacterial* suspension with a concentration of 1 × 10^5^ CFU/mL was added. After that, the shake tubes were incubated at 37 °C with agitation at 150 rpm for 24 h, and the absorbance of each shake tube at 600 nm (for *E.coli*) or 450 nm (for *S.aureus*) was finally measured by a microplate reader (Thermo Fisher Scientific Co., Waltham, MA, USA). The bacteria livability was calculated according to the following formula:

(2) $$\mathrm{Bacteria}\;\mathrm{livability}\;=\frac{A_{s}}{A_{c}}\times 100\%$$

where *A*_s_ is the total number of surviving bacteria in the experimental groups and *A*_c_ is the total number of surviving bacteria in the blank group without material.

In order to observe the inhibitory effect of series CNCs-based PUs on *E.coli* and *S.aureus* more vividly, the colony formation of *E.coli* and *S.aureus* after being treated with different materials for 24 h was observed. Specifically, 10 µL of the *bacterial* suspension from the above shake tubes was used to coat the plates, and after being incubated overnight at 37 °C, the colonies were photographed.

## 3. Results and Discussion

### 3.1. Structural Characteristics of CNCs-Based PUs

**Figure 2:**
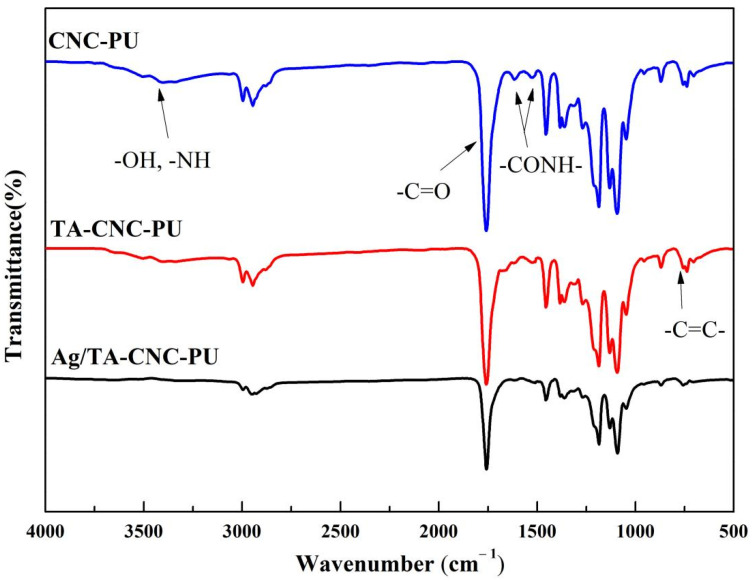
The FT-IR spectra of CNCs-based PUs.

The chemical structures of CNC-PU, TA-CNC-PU and Ag/CNC-TA-PU were investigated by FT-IR, and the resulting spectra were presented in Figure 2. From the spectra of CNC-PU and TA-CNC-PU nanocomposites, absorbance of around 3200–3550 cm^−1^ represented the stretching vibration of -OH in CNCs or TA, and -NH groups in PU [34], while the strengths of those peaks were weakened in Ag/CNC-TA-PU, which indicated that some of these functional groups were consumed. This phenomenon is consistent with the oxidation of trihydroxyphenyl and dihydroxyphenyl groups to their quinone forms during the redox reaction. In other words, TA acted both as reducing and stabilizing agents for the preparation of Ag/CNC-TA-PU [24]. Additionally, the sharp peaks at around 1750 cm^−1^ represent the typical C=O stretching vibration. Compared with CNC-PU, the bends at around 757 cm^−1^ were attributed to C=C distortion vibrations in the benzene of TA-CNC-PU [35], which indicated that TA was present in the TA-CNC-PU nanocomposites. In addition, the characteristic peaks at 1662 cm^−1^ and 1525 cm^−1^ of TA-CNC-PU and Ag/CNC-TA-PU, which corresponded to the absorption of carbonyl groups (C=O) and N-H bending deformation combined with C-N asymmetric stretching, respectively, also changed, which may be due to the formation of hydrogen bond between the -NHCOO- and TA. Thus, it can be inferred that three types of CNCs-based PUs have been successfully synthesized in this study.

**Figure 3:**
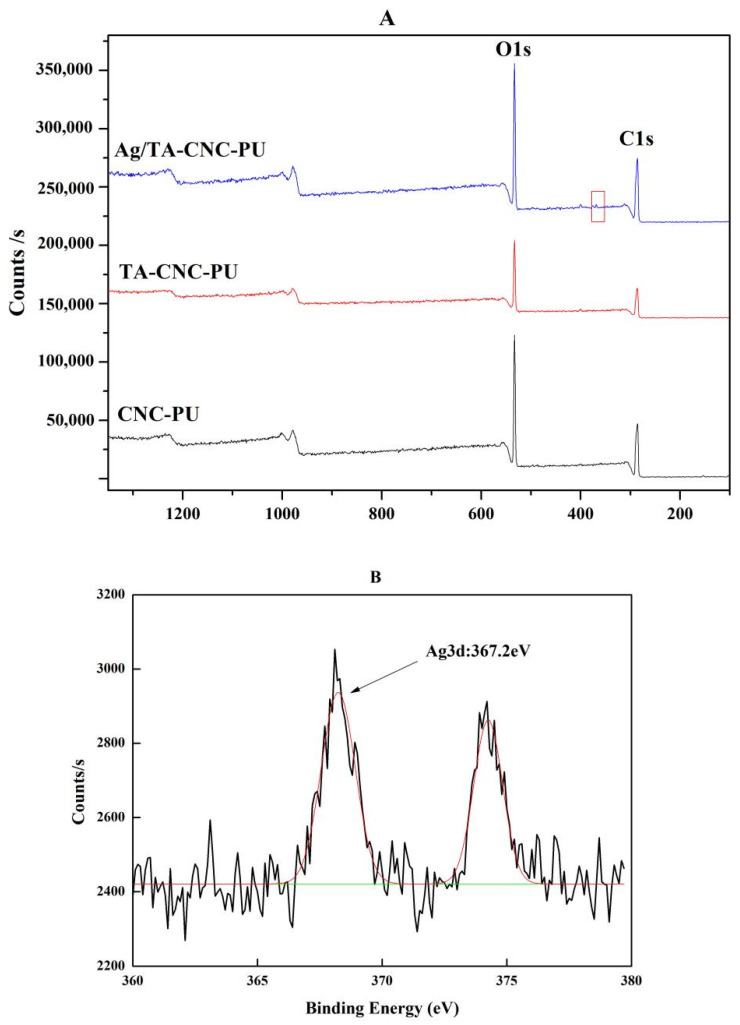
The XPS spectrum of CNCs-based PUs (**A**) and 3d core level in Ag/TA-CNC-PU (**B**).

To further confirm the modification of CNCs, XPS was used to determine element composition of series CNCs-based PUs, and the valence state of Ag in Ag/CNC-TA-PU were also investigated by auger electronic spectrum analysis, and the results are represented in Figure 3A,B. The XPS spectrum of series CNCs-based PUs showed the binding energies of two critical peaks, C1s (285 eV) and O1s (532 eV) for carbon and oxygen atoms of CNC, respectively [36]. Compared to CNC-PU and TA-CNC-PU, successful deposition of Ag NPs on the materials was illustrated by the presence of characteristic Ag 3d signals on the Ag/TA-CNC-PU (red box) XPS spectrum. Further analysis showed that the orbital binding energy of Ag 3d5/2 was 367.2 eV, which indicated that the synthesized Ag NPs in Ag/CNC-TA-PU were in their metallic forms [37]. Therefore, as expected, CNCs-based PUs, loaded with Ag NPs, were prepared successfully.

**Figure 4:**
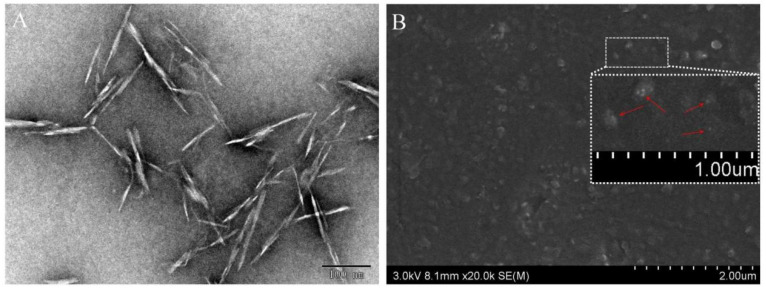
The TEM image of CNCs (**A**) and SEM images of Ag/TA-CNC-PU films (**B**).

The acid hydrolyzed freeze-dried CNCs showed long rod-like morphology (Figure 4A) with an average length of 100–200 nm and an average width of 10–20 nm, which was consistent with the data reported in the literature [37]. Meanwhile, the agglomeration phenomenon was also observed in the TEM images due to the formation of hydrogen bonds on the surface of CNCs. Figure 4B showed the surface morphology of Ag/ TA-CNC-PU nanocomposite films. From the picture, particles with a diameter of about 200 nm could be clearly identified in the matrix of the nanocomposite films, which corresponded to the CNCs in the surface of the nanocomposite films. In addition, as shown in Figure 4B, these CNC fillers were well dispersed in the PU matrix, implying that a good compatibility between the nanofillers and polymer matrix was achieved, which may be ascribed to the covalent bonding between CNCs and the molecular chains of PU. The inset displays the greater magnification of the surface of Ag/TA-CNC-PU nanocomposite films, and the white dots, with an average diameter of about 10 nm, could be clearly observed on the surface of CNC fillers, which corresponded to the Ag NPs.

### 3.2. Thermal Analysis

**Figure 5:**
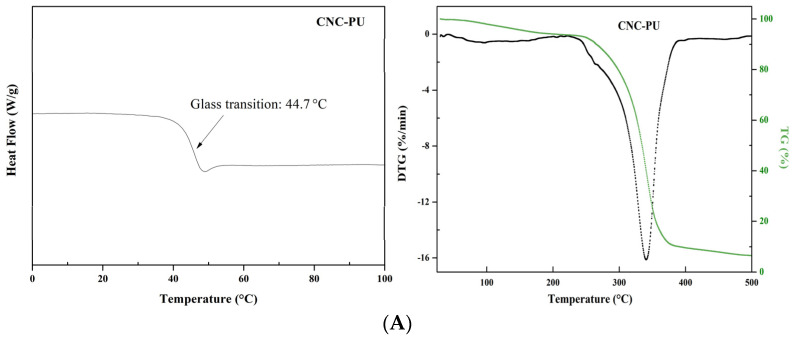

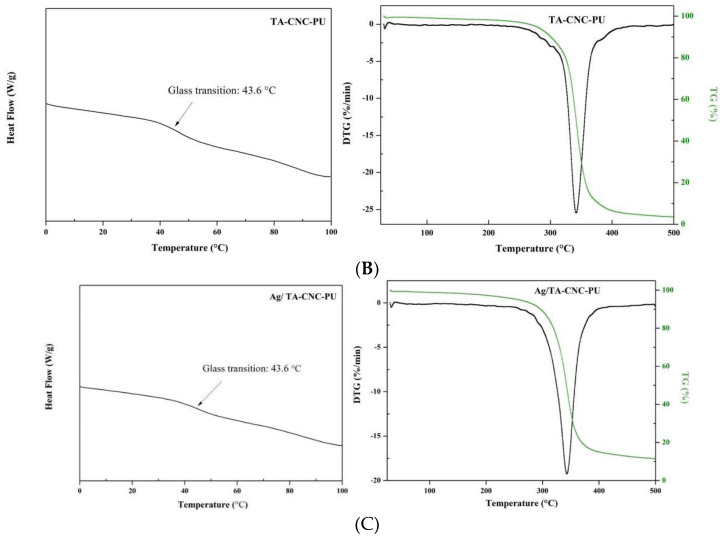
The DSC and TG/DTG curves of CNC-PU (**A**), TA-CNC-PU (**B**) and Ag/TA-CNC-PU (**C**).

To compare the thermal degradation behavior of series CNCs-based PUs, the materials needed to be dried in a similar manner prior to TGA and DSC experiments, and the DSC and TG/DTG measurement results were shown in Figure 5. An obvious glass transition temperature (T_g_) at 44.7 °C of CNC-PU nanocomposites was observed, the T_g_ of TA-CNC-PU and Ag/TA-CNC-PU was lower when compared to CNC-PU, which could be attributed to a decreased crystallinity of CNC and PU after linking by the TA and Ag NPs. The observed mass loss first occurred at around 100 °C and was more prominent in CNC-PU in comparison to TA-CNC-PU and Ag/TA-CNC-PU. This mass loss probably arose due to the evaporation of adsorbed water. As can be seen from the figure, all series of CNCs-based PUs had only one weight loss temperature zone. The onset (T_onset_) degradation temperature for CNC-PU was about 245 °C, which was lower than that of TA-CNC-PU and Ag/TA-CNC-PU, while the maximum degradation temperatures (T_max_) for CNC-PU, TA-CNC-PU and Ag/TA-CNC-PU were relatively close, all at about 340 °C. It can therefore be seen that the thermal stability was significantly improved in TA-CNC-PU and Ag/TA-CNC-PU as compared to CNC-PU. This probably because TA could be firmly grafted onto the CNCs of CNC-PU through covalent bonding under the action of aldehyde and its phenolic hydroxyl could form a hydrogen bond with the segments such as PEG or -NHCOO-, so as to improve the thermal stability of the obtained materials. In addition, the ortho phenolic hydroxyl group in TA could also form a stable five-member chelate ring with Ag^+^, thus immobilizing Ag^+^ in the material without affecting its thermal stability.

### 3.3. Hydrophilic Properties

**Figure 6:**
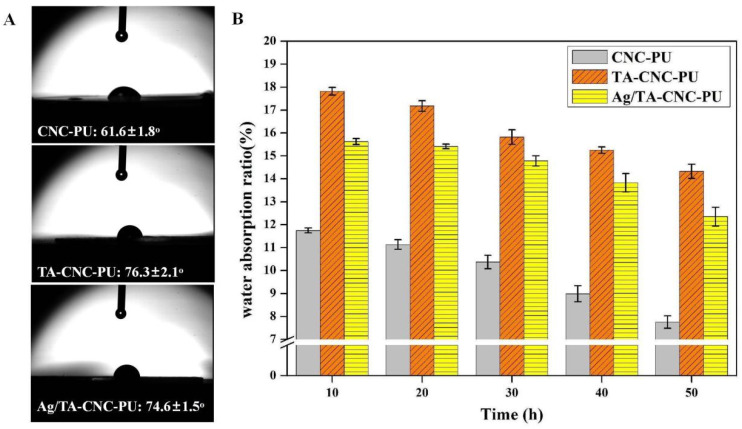
The hydrophilic properties of CNCs-based PUs. (**A**) Static water contact angle; (**B**) Water absorption ratio.

The water contact angle on the surface of the material can reflect the instantaneous hydrophilicity and hydrophobicity of water drops onto the material surface. The larger the contact angle is, the stronger the hydrophobicity is, and vice versa. Figure 6A showed the water contact angle analysis of series CNCs-based PUs. It can be observed from the figure that all three types of CNCs-based PUs were hydrophilic (held a contact angle less than 90°) due to the fact that CNCs are inherently hydrophilic materials with a low contact angle of about 25.6° [38], and thus these CNCs-based PUs could be further applied as water-driven biomaterials. One more interesting thing was that, as TA has high hydrophilicity, a slight increase in contact angle to 76.3 ± 2.1° was observed after TA was introduced into the molecular structure of CNC-PU. It was speculated that the material surface conformation changes and hydrophilic site occlusion during film formation may play an important role. Specifically, TA is insoluble in CHCl_3_ and interacted easily with the hydrophilic sites (including -NHCOO- in a PU hard segment, -C-O-C- in a PU soft segment, and -OH functional groups in CNCs) in PU, so during the film formation process, it may be hidden inside the film, causing the relatively hydrophobic PU to gather on the surface of the film. On the one hand, the introduction of TA into the material would increase the surface roughness of the film [38], which would also lead to an increase in the water contact angle of the film. When compared Ag/TA-CNC-PU with TA-CNC-PU, the hydrophilic property of Ag/TA-CNC-PU was slightly enhanced. This might be due to the strong binding ability of Ag NPs to TA, which exposed part of the hydrophilic segments in CNC-PU that were originally bound to TA. The water absorption rate of the material reflects the hydrophilicity and hydrophobicity of the material as a whole, when in contact with water for a long time. The higher the water absorption rate is, the better the hydrophilicity is. PU suffers from poor moisture barrier properties because of the residual polar groups on the cross-linked structure. These functional groups allow for the association with water molecules and the water is able to penetrate through the matrix that eventually affect its properties [17]. Figure 6B showed the water absorption for series CNCs-based PUs over time. Compared with CNC-PU, the water absorption of TA-CNC-PU nanocomposites has increased. This result was consistent with past studies [39,40]. One factor that could contribute to this increase in the water absorption of the TA-CNC-PU nanocomposites is the structural change and amount of polar functional group exposure in the polymer matrix. Specifically, after introducing TA to CNC-PU, some -OH groups on the surface of CNC were converted to abundant hydrophilic Ar-OH groups. However, these Ar-OH groups easily form hydrogen bonds with various groups, such as hydrophilic -OH groups in CNC and -NHCOO- groups in PU, so that the hydrophilic sites of TA-CNC-PUs were hidden during the film formation process. When the membrane was in full contact with water, the H_2_O molecules competed with Ar-OH, resulting in the exposure of hydrophilic sites in the material. Such changes in the composition and structure of the surface and interior of TA-CNC-PU nanocomposites contributed to the scaling up from trapping water molecules and permeation into the nanocomposite matrix. For Ag/TA-CNC-PU nanocomposites, a lower water uptake was observed when compared to TA-CNC-PU. This might be due to the consumption of some Ar-OH groups in TA by Ag NPs during the reduction process [24]. In addition, it was also observed from the figure that with the prolongation of soaking time, the water absorption of serial CNCs-based PUs showed a slow downward trend, which was considered to be the simple degradation of hydrophilic materials.

### 3.4. Antibacterial Activity

**Figure 7:**
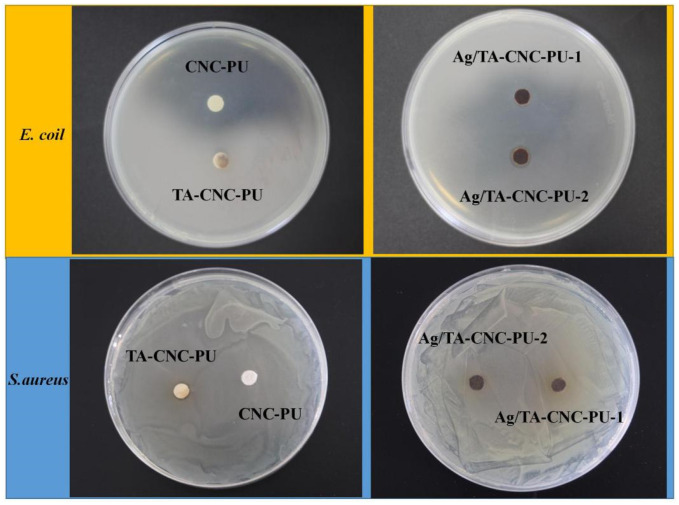
The antibacterial activity of CNCs-based PUs against *E. coli* and *S.aureus*.

Ag NPs are broad spectrum antimicrobial agents and could inhibit the growth of a wide range of bacteria [24,41]. Studies have also shown that TA has an inhibitory effect on certain microorganisms [42]. So, the contact-killing performance of the CNC-PU, TA-CNC-PU and Ag/TA-CNC-PU was assayed by the inhibition zone test (Figure 7). In the section, *E. coli* and *S.aureus* were spread on the LB agar plates and then placed in contact with the materials. However, an interesting phenomenon was observed in this experiment. For *E. coli*, due to the strong antibacterial property of Ag NPs, *E. coli* growth was inhibited in the Ag/TA-CNC-PU experience groups and here also showed an Ag NPs concentration dependence, while the CNC-PU and TA-CNC-PU did not display any activity against *E. coli*. Specifically, Ag/TA-CNC-PU-1 means that the mass ratio of TA to CNC-PU added in the grafting reaction was 0.3, and the mass ratio of AgNO_3_ to TA0.3-CNC-PU was 2%, while Ag/TA-CNC-PU-2 means that the mass ratio of AgNO_3_ to TA0.3-CNC-PU was 5%. When comparing the size of bacteriostatic circle between the two, it can be found that the bacteriostatic circle diameter of Ag/TA-CNC-PU-2 was significantly larger than that of Ag/TA-CNC-PU-1, which means Ag/TA-CNC-PU-2 showed a better bacteriostatic effect. For *S.aureus*, a bacteriostatic circle was observed around TA-CNC-PU, but there were no obvious inhibition zones around the CNC-PU and Ag/TA-CNC-PU experience groups. Accordingly, Ag/TA-CNC-PU had a better inhibitory effect on *E.coli*, while TA-CNC-PU had better inhibitory effect on *S.aureus*. These results were consistent with those reported in the literature [42,43]. Furthermore, according to the literature, Ag NPs damages some of the basic cell functions of bacteria and destroys its surface structure, while TA disrupts cell wall integrity by binding to the peptidoglycan of *S. aureus* cell walls [44,45]. However, the inhibition zone diameters of TA-CNC-PU and Ag/TA-CNC-PU were smaller than those of pure TA and Ag NPs in the literature, which may be because that TA and Ag NPs were connected to CNC-PU in the form of covalent binding according to the above experimental results, so they can not be dissolved to play an antibacterial role. To verify this speculation, further antibacterial testing of series CNCs-based PUs was carried out.

**Figure 8:**
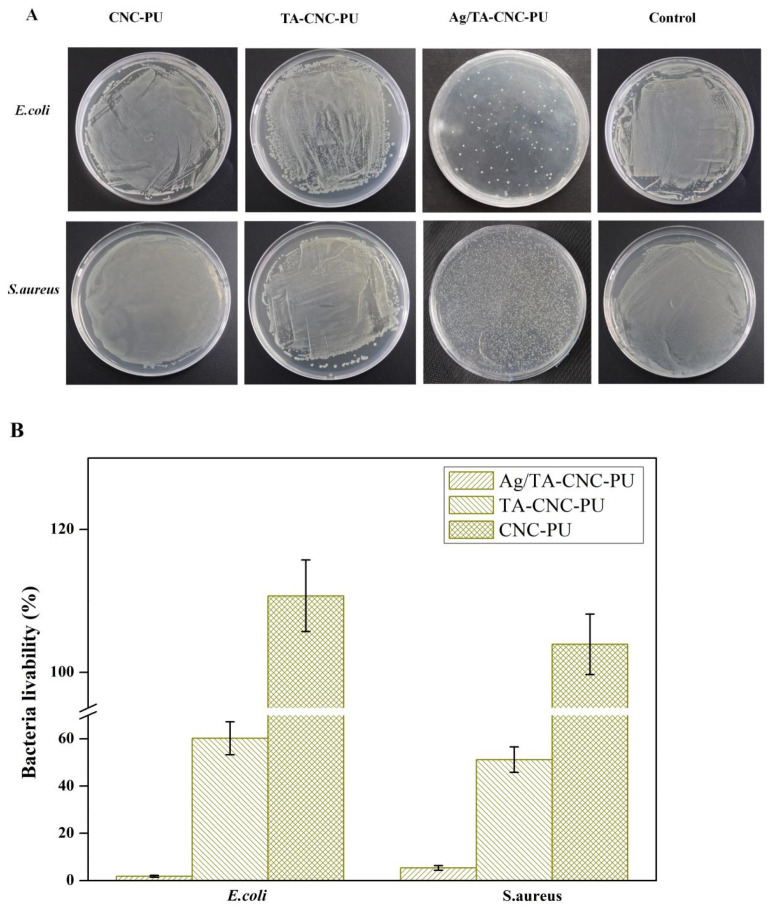
Inhibition of CNCs-based PUs against *E. coli* and *S. aureus*. (**A**) Colony growth, (**B**)  bacteria livability.

Figure 8A,B show the inhibitory effect of series CNCs-based PU materials on *bacteria*. From the figure it can be seen that Ag/TA-CNC-PU-2 nanocomposite material had the best antibacterial effect when compared to CNC-PU and TA-CNC-PU. When exposed to bacteria for 24 h, the CNC-PU groups not only had no inhibitory effect on *E.coli* and *S.aureus*, but also showed a certain promoting effect, which may be attributed to their good cytocompatibility [14]. However, after loading Ag NPs, the obtained Ag/TA-CNC-PU-2 nanocomposite material was able to inhibit 98.17% of *E.coli* and 94.65% of *S. aureus*. This may be because Ag NPs could not only combine negatively charged anionic groups, such as -COO^−^ and -O-PO^3−^, on the cell wall, but also interacted with proteins in the outer membrane of *E. coli*, so Ag/ TA-CNC-PU-2 showed stronger antibacterial properties against *E.coli*. For TA-CNC-PU groups, when compared to CNC-PU, they showed a certain ability to inhibit the growth of *E. coli* and *S. aureus* after 24 h. This might be due to the fact that TA gradually played a major role in inhibiting *bacteria* growth [40,43]. To summarize, CNC-PU materials could promote the growth of bacteria and TA-CNC-PU could inhibit the activity of bacteria to a certain extent, while, finally, Ag/TA-CNC-PU-2 showed better antibacterial properties due to the synergistic effect of TA and Ag NPs contained in its structure (TA was able to inhibit the growth of bacteria and Ag NPs were able to effectively kill bacteria).

## 4. Conclusions

In summary, we successfully prepared serious types of antibacterial nanocomposite networks with the triblock copolymer PLLA-PEG-PLLA as the soft segment, CNC nanofillers as cross-linkers, and further covalently bound TA and deposited Ag NPs. FT-IR, XPS, TEM and SEM corroborate the successful modification of TA and Ag NPs. The introduction of TA and Ag NPs could significantly improve the thermal stability and antibacterial properties of the nanocomposite network. Specifically, their antibacterial properties could be controlled by adjusting the content of TA and loaded Ag NPs to satisfy different biomedical application demands. Simultaneously, all the three kinds of CNCs-based PU nanocomposites exhibited excellent hydrophilic properties. Therefore, the water–responsive shape–memory nanocomposite networks which were prepared in this study could potentially be applied in the biomedical fields in various capacities, such as self-tightening sutures and adaptive smart dressing with good antibacterial properties.

## Data Availability

Not applicable.
